# Facile Method for Fabricating Microfluidic Chip Integrated with Microwell Arrays for Cell Trapping

**DOI:** 10.3390/mi10110719

**Published:** 2019-10-25

**Authors:** Hongyue Wu, Zhixing Ge, Wenguang Yang, Xiaoduo Wang, Xiaodong Wang, Haibo Yu

**Affiliations:** 1 School of Mechanical Engineering and Automation, Northeastern University, Shenyang 110004, China; wuhongyue_neu@139.com (H.W.); xdwang@mail.neu.edu.cn (X.W.); 2 State Key Laboratory of Robotics, Shenyang Institute of Automation, Chinese Academy of Sciences, Shenyang 110016, China; gezhixing@sia.cn (Z.G.); wangxiaoduo@sia.cn (X.W.); 3 University of the Chinese Academy of Sciences, Beijing 100049, China; 4 School of Electromechanical and Automotive Engineering, Yantai University, Yantai 264005, China; ytu_yangwg@163.com

**Keywords:** microstructure fabrication, microwell arrays, cell capture, microfluidic chip

## Abstract

With the development of biomedical technology, personalized diagnosis and treatment at the single-cell level are becoming more important in the medical field. As one of the most powerful tools, microfluidic chips have shown significant potential for various applications related to cell separation, cell proliferation, and cell behavior analysis. However, fabricating microfluidic devices requires complicated procedures and high-cost equipment. In this study, an optofluidic maskless lithography method was proposed for rapid fabrication of microfluidic devices integrated with microwells. Through the use of this approach, microwells can be on-line designed and the exposure patterns can be modulated. Single or multi polystyrene microspheres were successfully trapped by using the designed microwells. The capture of MCF-7 cells and cell arrays indicated that the microfluidic devices fabricated in the present study can be applied for cell research.

## 1. Introduction

Owing to the developments in biological fields, personalized diagnoses and treatments at the single cell level have become important, which will introduce new potential breakthroughs for biomedicine [1,2,3,4]. Recently, the early detection of cancer and drug action on cancer have attracted considerable attention in the field of biotechnology. Microfluidic device is a useful tool in cell research with advantages such as high-throughput manipulation, high level of integration, and requirement of less time and fewer reagents [5,6,7,8,9]. Furthermore, microfluidic devices have broad applications in cancer cell trapping. Microwell arrays in microfluidic device are often used to trap cells and construct cell arrays [10,11].

Cells trapped in a microfluidic device can be cultured, used to detect markers, manipulated, and employed for drug screening. Rettig et al. have developed an easy-to-use method using microwell array fabricated through soft lithography for a single cell trap. Anchorage-dependent cell (3T3 fibroblast cells) and anchorage-independent cell (Basophilic leukemia cell) were successfully trapped in microwell arrays [12]. Lin et al. improved the traditional structure of microwells and introduced a double-well design, including a tapping well and a culturing well for single cell array construction. Neural stem cell differentiation and A549 cell proliferation were performed using this device [13]. Assal constructed a 3D microwell array using polyethylene glycol (PEG) solution cured by ultraviolet (UV) light based on a polydimethylsiloxane (PDMS) mold. Human B cells infected by Kaposi’s sarcoma-associated herpesvirus (KSHV) were trapped in these microwells for 3D culture [14].

Microfluidic device has been applied in various fields for cell separation, cell proliferation, and cell behavior in biology research [15,16,17]. In particular, it has received attention for the in situ characterization of cells after trapping them using microfluidic device equipped with microwell arrays [18,19]. There are traditional methods for fabricating microfluidic devices such as soft lithography, photolithography, and laser direct writing [20,21,22]. Although photolithography has the advantages of high resolution, a physical mask is essential during the fabrication process, and this technique requires extreme environmental conditions. Soft lithography has been successfully and widely applied to microfluidic device fabrication. While it is an easy-to-use approach, it requires a pre-designed mold or stamp, making it considerably time-consuming. Laser direct writing can fabricate complicated 3D microstructures without the need of photomasks owing to its high-resolution and flexibility. However, the linear scanning mode is time-consuming. Therefore, tedious procedures and high-cost equipment are the main challenges of the current methods for fabricating microfluidic devices. Thus, it is crucial to develop a facile technique for creating microfluidic devices that can satisfy the requirements of cell separation and cell trapping.

In this paper, we present optofluidic maskless lithography for rapid fabrication of microfluidic devices [23]. Poly(ethylene glycol) diacrylate (PEGDMA) was selected as the material for microstructures fabrication, and the cured microstructures were firmly adhered to the substrate. The structures of microfluidic chips were on-line designed, and the exposure patterns were modulated by digital micromirror device (DMD). Compared with traditional methods, the proposed system is more flexible and cost-efficient. Furthermore, microwell arrays with various shapes and sizes can be constructed on the microfluidic chip.

## 2. Materials and Methods

### 2.1. Experimental Setup

A charge coupled device (CCD) camera (DAHENG IMAGE, Inc. DH-SV2001FC, Beijing, China), a digital micrometer device (DMD) (Texas Instruments, Inc., Dallas, TX, USA), projection optical devices (Figure S1), and a computer are the main components of our optofluidic maskless lithography system. The computer changes the patterns flexibly, and they are projected onto the chip using the DMD. The cured arrays were captured by using the CCD camera. A UV laser, with a wavelength of 365 nm and maximum power of 53.33 mW/cm^2^, was used in the experiments. A microscope objective (LEICA, Inc. VZ105, Varga Hector, Switzerland) with a magnification factor of 140 and numerical aperture (NA) value of 0.28, was used. During the experiments, the curing time was set to 5 s, and the average depth of the microwell arrays is approximately 30 μm. The microfluidic chip is composed of two pieces of glass and layers of dual adhesive tape. To connect the hoses, two holes were punched in the top glass layer by using the engraving machine.

### 2.2. Materials

PEGDMA (Mn 750, Sigma Aldrich, St. Louis, MO, USA) is a chemical substance commonly used in food and medical and health gel materials. In this experiment, PEGDMA was selected as a photo-curable polymer, and diphenyl (2,4,6-trimethylbenzoyl)-phosphine oxide (TPO) was selected as the photoinitiator. TPO and PEGDMA were dissolved and stirred in ethyl alcohol for half an hour, and the final concentrations were 30% and 0.5 wt %, respectively. The entire process was carried out in a sterile and light-shielded environment.

According to previous studies, the shape of a microwell has significant influence on the fluid flow and alters the force acting on the particles [12,13,24]. Using microwell arrays to capture the cells depends on the force in the stream field. Microwell arrays of different shapes and sizes were manufactured by using the proposed system (Figure S2 and Figure S3).

### 2.3. Cell Culture

We used RPMI-1640 medium (HyClone, Logan, UT, USA) to culture MCF-7 breast cancer cells. Moreover, 1% penicillin/streptomycin and 10% fetal bovine serum were added in the medium. The temperature and CO_2_ content of the cell incubator were maintained at 37 °C and 5%, respectively. To obtain cell suspensions, MCF-7 cells were treated with trypsin for 3 min, and the cells attached to the bottom of the petri dish were blown using a pipette.

### 2.4. Fluorescent Staining

To demonstrate the effect of microwell arrays on capturing the breast cells, we carried out live/dead staining on the cells. We mixed 15-µL propidium iodide (PI), 10-µL Calcein-AM, and 7-mL phosphate buffer saline (PBS) to form a dye solution. The cells were treated with the dye solution and placed in a cell incubator for 15 min. Next, the dye solution was replaced by PBS. The eclipse Ti microscope (TIE, Nikon, Tokyo, Japan) comprises a platform, lens, camera head, control device, and cell incubator chamber. The fluorescence images of the captured cells were photographed by TIE. A scanning electron microscope (EVO MA10, Zeiss, Inc., Jena, Germany) was used to characterize the structures of the microwell arrays.

## 3. Result and Discussion

**Figure 1:**
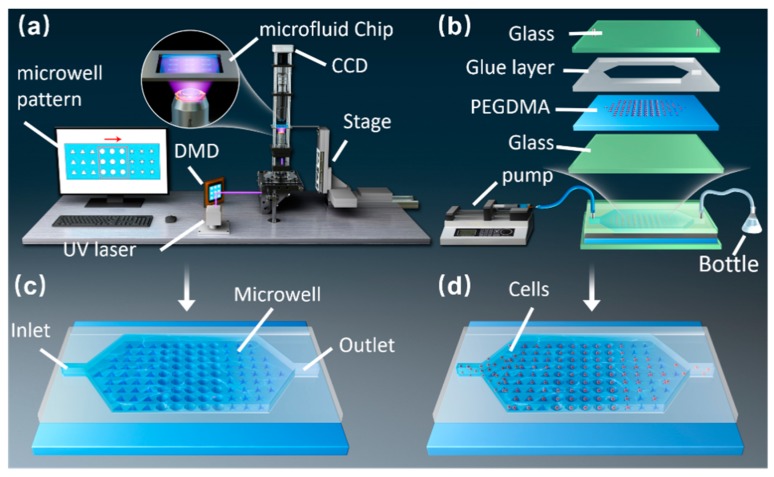
(**a**) Schematic of optofluidic maskless lithography system. (**b**) Composition and assembly of the chip. (**c**) System-based fabrication of microwell arrays. (**d**) Diagram of cell injection and capture.

Figure 1a shows the schematic of the optofluidic maskless lithography system that consists of a CCD camera, a DMD, projection optical devices, and a computer. The microwell arrays can be manufactured by changing the patterns of the DMD in real time. Figure 1b depicts the composition and assembly of the chip. During the experiment, we injected PEGDMA hydrogel into the microfluidic chip by using an injection pump. Once the liquid level of the hydrogel stabilized, the microwell arrays were cured using UV light (Figure 1c). After fabricating the arrays, absolute ethanol was injected into the chip to remove excess hydrogel. Next, the particles and cell suspensions were injected into the microfluidic chip (Figure 1d). Finally, the remaining particles and cells were washed with deionized water.

**Figure 2:**
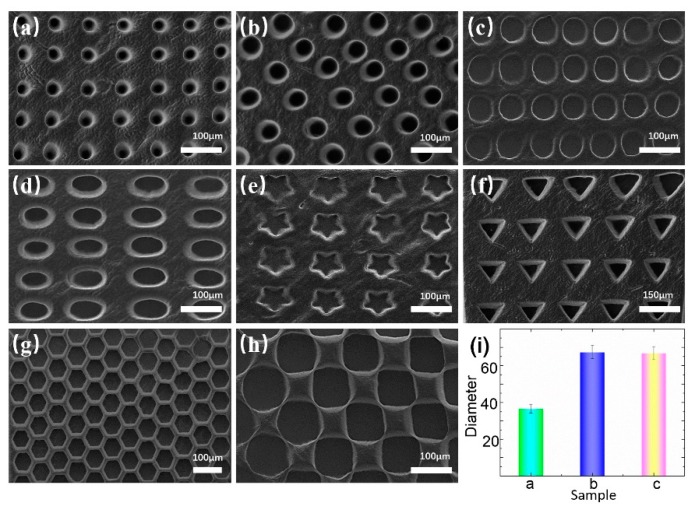
Characterizing microwell arrays via scanning electron microscope. (**a**–**c**) Circular microwell arrays with different sizes. (**d**–**h**) Microwell arrays with other patterns. (**i**) Diameter and standard deviation of circular microwell arrays. Scale bar: 100 µm.

The arrays exhibited high fidelity and uniformity. Circular, elliptical, and polygonal microwell arrays, with sizes ranging from 30–150 µm, were fabricated. Figure 2a–c shows the circular microwell arrays of different sizes. Figure 2d–h present the microwell arrays with other patterns, which demonstrates the flexibility and high precision of our system. The diameters and standard deviations of the circular microwell arrays are shown in Figure 2i. The statistical results show that the arrays are suitable for cell capture.

**Figure 3:**
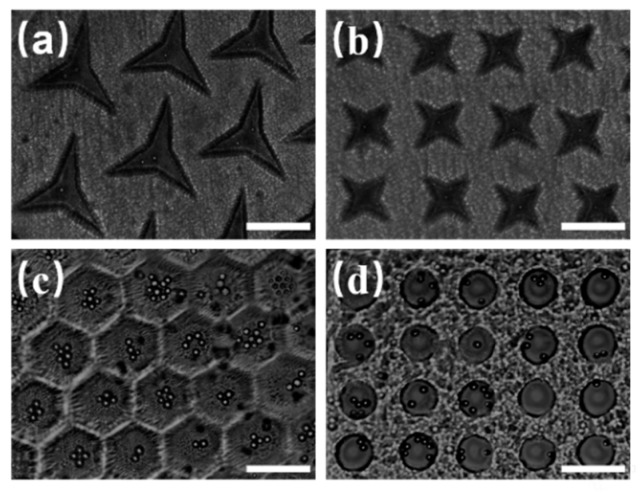
Polystyrene microspheres captured in microwell arrays with different shapes. The patterns are: (**a**) triangular, (**b**) quadrangular, (**c**) honeycomb, and (**d**) circular. Scale bar: 100 µm.

According to literature, the shape of microwells in micro-fluidic chips fabricated by PDMS plays a significant role in the capturing of microspheres and cells [25,26]. Here, we fabricated microwell arrays of different shapes using the PEGDMA in the optofluidic maskless lithography system. The microwell array was used to capture polystyrene (PS) microspheres with diameter of 10 µm, initial concentration of 1.8 × 10^6^ /mL. The influence of the shape of microwells on microspheres capturing was analyzed. Figure 3 shows microwell arrays of different shapes fabricated by the light-induced integrated system, which were used to capture the polystyrene microspheres. The triangular microwells shown in Figure 3a can capture an individual particle. Meanwhile, the trapped particle located in the center of the triangular microwells. Similarly, the quadrangular microwell arrays can capture individual particles in the center of each microwell, as shown in Figure 3b. In Figure 3c, the number of particles in each honeycomb microwell increases with an increase in the concentration of microspheres suspension. The same results can be obtained with circular microwells, as shown in Figure 3d. While all four kinds of microwell arrays could capture microspheres, it was found that the circular microwell arrays exhibited the best homogeneity, and their size after curing was the same as the designed size. In addition, the probability of capturing microsphere in all the experiments based on the circular microwells was 85% or higher. Therefore, they were adopted for subsequent optimization experiments.

**Figure 4:**
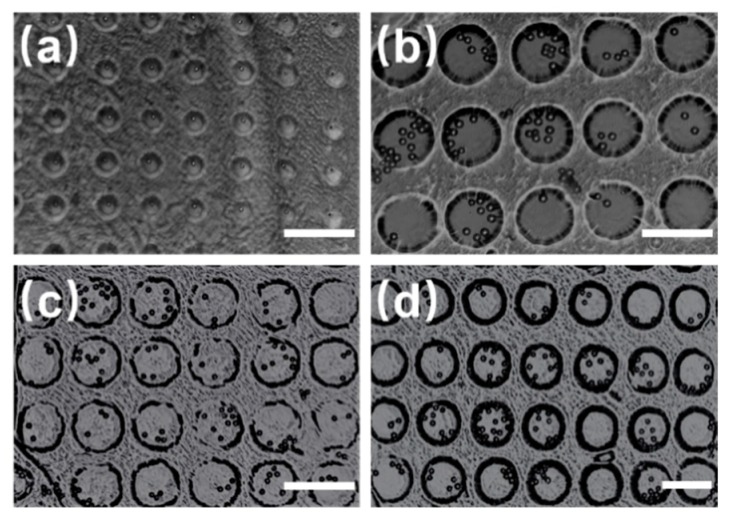
Microspheres captured in microwell arrays. The diameters are: (**a**) 30 µm, (**b**) 50 µm, (**c**) 70 µm, and (**d**) 80 µm. Scale bar: 100 µm.

To demonstrate the influence of the diameter of the circular microwells on the captured microsphere, microwell arrays with diameters of 30 µm, 50 µm, 70 µm, and 80 µm were fabricated, as shown in Figure 4. All the microspheres used are polystyrene microspheres with a diameter of 10 µm. When the diameter of the microwells was 30 µm, the captured individual microspheres were located at the center of the microwells. Further, when the diameters were 50 µm, 70 µm, and 80 µm, several microspheres were captured and most of them were located at the center of the microwells. In conclusion, when the diameter of the PS microsphere tended to close to that of the microwells, the easier it was to capture an individual particle in the center of the microwells. Further, the number of the microspheres increased with the diameter of the microwells.

**Figure 5:**
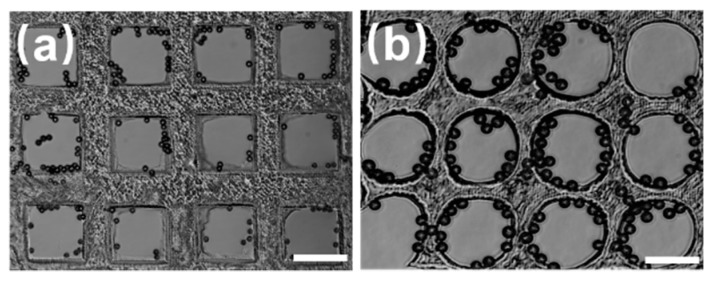
Microspheres captured in shallow arrays of the microwell. The patterns are: (**a**) square and (**b**) circular. Scale bar: 100 µm.

When the curing time was short during the fabrication of the microwell arrays, their depth would be moderately shallow, making them adverse for capturing the microspheres. The microspheres captured by the shallow microwells were generally located along the boundary of the microwells and tend to flow out of the microwells when deionized water was pumped into the microfluidic chip. In this situation, the captured microspheres were induced by fluid shear stress, Saffman lift, and Stokes drag simultaneously. The combined effect of these forces made microspheres flow toward the boundary of the microwells; they easily flowed out along with the deionized water. Figure 5 shows that the captured microspheres located near the boundary of the circular and rectangular microwells when the microwell array is shallow tend to flow out when deionized water is pumped in again. In conclusion, the parameters of the microwells had a considerable influence on the capture results of the PS microspheres.

**Figure 6:**
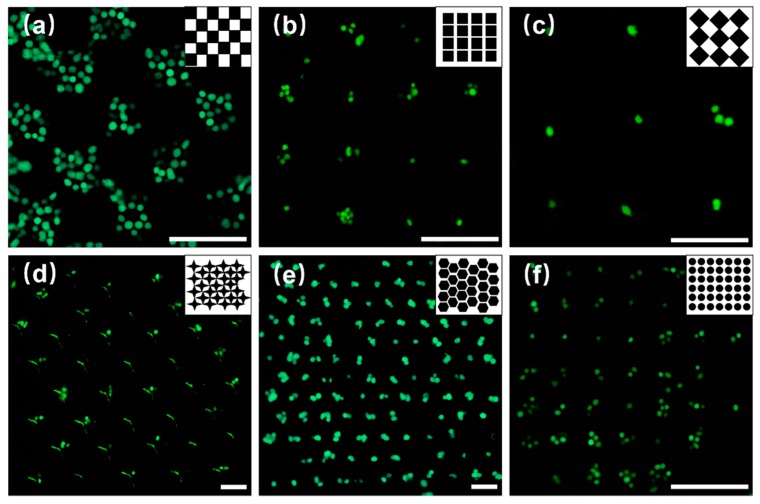
Breast cancer cells captured by microwell arrays with different shapes. (**a**) Chessboard, (**b**) Rectangular, (**c**) Diamond, (**d**) Four-angle star, (**e**) Honeycomb, and (**f**) Circular. Scale bar: 150 µm.

Based on the experimental results of capturing PS microspheres, we performed experiments to capture breast cancer cells. Owing to the disadvantages of the shallow microwell arrays, microwells with a depth higher than 15 µm were fabricated in the subsequent experiments. Figure 6a,b shows that when the concentration of the cell is 7.5 × 10^5^ /mL, the microwell array was almost full of cells, and when the concentration of the cell is 2.5 × 10^5^ /mL, the number of the cells in the microwell array evidently decreased. Figure 6c shows that when the concentration of the cell was attenuated by three times (1.25 × 10^5^ /mL), individual cells could be captured. The experimental results show that the size of the rectangular microwells (Figure 6b) was considerably large to capture cells, and 2.5 × 10^5^ /mL was the suitable cell concentration that should be selected to enhance the capture efficiency. Figure 6d–f shows the cell capture results using microwell arrays shaped as a four-angle star, honeycomb, and circular. All the arrays could capture individual (Figure 6d) and multiple cells (Figure 6e,f), and most of the captured cells were located at the center of the microwells.

## 4. Theoretical Analysis

**Figure 7:**
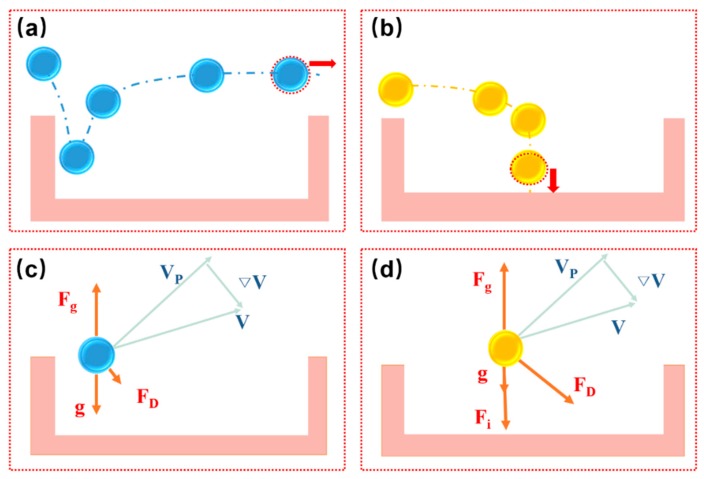
Schematic of particles flowing through the microwell. (**a**) Particle flowing through the microwell. (**b**) Particle captured by the microwell. (**c**,**d**) Diagrams of the forces acting on a particle.

As shown in Figure 7, there are two situations when particles move above the microwell array. One portion of the microparticles tends to move towards the edge of the microwells, whereas the other portion of the microparticles will probably be deposited at the bottom of the microwells. It is essential to determine which forces dominate this movement of the microparticles. Considering the buoyancy force, the gravity force of the microparticles can be considered to be negligible. When particles enter near the edge of the microwell, the Stokes forces are small; therefore, particles are less likely to fall to the bottom. As the particles enter the middle of the microwell, the particles are subjected to additional mass forces, and the Stokes force also increases. Under the influence of the resultant force, the particles easily fall to the bottom of the microwell. The Stokes force associated with the size of the microparticles and the viscous coefficient of the fluid on the particles cannot be neglected as they influence the final velocity of the microparticles under a fluidic condition. When the microparticles enter the region near the sidewall, they are subjected to a shear force that lifts the particles. This force is called Saffman force. In particular, when the microwell is shallow, the Saffman force acting on the particles in the microwell needs to be considered [24]. The resultant force exerted on the particles in microwells is significantly affected by the flow field. Based on the abovementioned analysis, we performed a flow field simulation using COMSOL (COMSOL Inc., Burlington, MA, USA), which is a multiphysics simulation software.

From the brief discussion above, we can learn that the force and motion of the microparticles in microwell arrays are dominated by the fluidic motion. Based on this, we simulated the flow field of microwell arrays with different shapes in the above experiments to study the hydrodynamic characteristics of microparticles under fluidic conditions [24]. We used COMSOL to simulate the flow field and selected the laminar flow model to attain a unidirectional flow. Based on the principle of mass conservation, the fluid density at any point in the flow field satisfies the continuity equation. For steady flow,

(1) ∂/∂t=0

The equation of continuity is

(2) ∇·(ρV)=0

where *ρ* is the density of the fluid, and *V* is the velocity vector of the fluid.

In microfluidic chips, the flow of fluids is generally similar to that of incompressible isotropic Newtonian fluid with constant density and viscosity. The N–S equation is simplified as follows:

(3) $$\frac{\partial V}{\partial t}+\left(V\cdot \nabla \right)V=-\frac{1}{\rho }\nabla p+\mu \nabla^{2}V+f_{\vartheta }$$

where *P* is the pressure; *µ* is the dynamic viscosity; and $f_{\vartheta }$ is the volume force per unit mass fluid.

**Figure 8:**
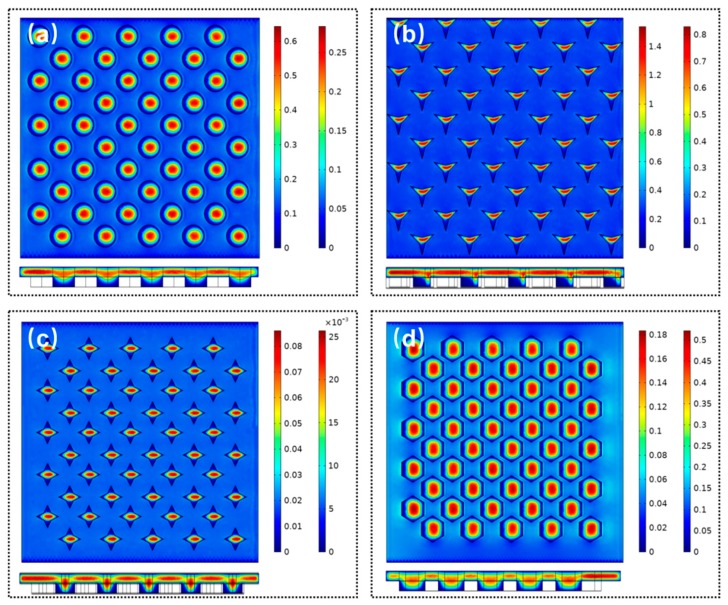
Distribution of the fluid field of different microwell arrays. (**a**) circle, (**b**) triangle star, (**c**) diamond, and (**d**) honeycomb.

We first analyzed the influence of the geometry shapes of microwell array as shown in Figure 8; four microwell shapes were adopted, i.e., circle, triangle star, diamond, and honeycomb. The depth of all microwell was set to 40 µm. For all types of microwell structures, the maximum fluidic velocity appears at the center of the microwell. The minimum fluidic velocity is attained at the bottom of the microwell. For the triangular microwell, it seems to be non-symmetric. The fluidic velocity appears to be minimum around the top edge of the sidewall of the microwells. This is because the direction of the flow field will vary around the top edge of the microwells. Meanwhile, the fluid inside the microwell needs to be excluded. Therefore, the magnitude of the fluidic velocity will initially decrease and then increase above the microwells. The trend will be inverted when the fluid flows through the center of the microwells.

**Figure 9:**
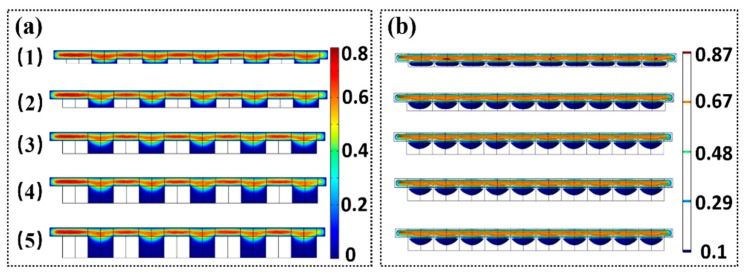
Cross section of flow field. (**a**) Flow field simulation results of the circular microwell arrays with radius of 30 µm and heights of 10, 20, 30, 40, and 50 µm. (**b**) Velocity contour distribution of the microwell flow field.

The experiments indicate that the depth of the microwells has a major effect on the amount of microparticles or cells trapped. Evidently, it is difficult to trap the microparticles when the depth of microwell is extremely small. The microparticles will move along the direction of the fluid flow. In contrast, several microparticles will be trapped if the depth of the microwells is extremely large. To observe the variation of fluidic velocity along the vertical direction of the microwell, we used the circular microwell to conduct the simulation of the flow field. The depth of the microwell varied from 10 µm to 50 µm. Figure 9 shows that when the depth of the microwell reaches a magnitude, the velocity of the middle and lower parts of the micro-pit is almost zero. The increase in the depth of the microwell has slight effect on the flow velocity. The simulation indicates that the optimized depth of the microwell is approximately 30 µm, which is in accordance with the experimental results.

## 5. Conclusions

In this study, the optofluidic maskless lithography method was used to fabricate microfluidic chips integrated with microwell arrays. It was demonstrated that maskless lithography was a versatile approach capable of developing customized microfluidic chips. PEGDMA hydrogel was used as the material for microstructure fabrication due to its good biological compatibility. In the microfluidic chips, microwell arrays of various shapes and sizes were constructed and optimized. The experimental results show that the shape and depth of the microwell has a significant influence on the process of trapping microspheres. Finally, MCF-7 cells were captured, and the cell arrays were formed, indicating that the microfluidic devices fabricated by using the proposed system exhibits potential for applications pertaining to cell research.

## Supplementary Materials

The following are available online at https://www.mdpi.com/2072-666X/10/11/719/s1, Figure S1: schematic of digital micromirrors, Figure S2: relation between deepness of the microwells and exposure time, and Figure S3: SEM image of microwell array.
