# Supplementary material for: Facile Method for Fabricating Microfluidic Chip Integrated with Microwell Arrays for Cell Trapping
Source: Micromachines (Basel). 2019 Oct 25;10(11):719. doi: 10.3390/mi10110719 (PMC6915356; doi:10.3390/mi10110719)
Supplement: Supplementary file 1 [file micromachines-10-00719-s001.pdf]

micromachines

Supplemental Materials

Facile Method for Fabricating Microfluidic Chip Integrated with Microwell Arrays for Cell Trapping

Hongyue Wu [1,2], Zhixing Ge [2,3], Wenguang Yang [4], Xiaoduo Wang [2], Xiaodong Wang [1] and Haibo Yu [2,*]

1. Details of the Experimental Setup

The experimental system can be divided into five parts: ultraviolet laser (wavelength: 375 nm, power modulation range: 0–50 mW), digital micromirror device (DMD), projection optics, 3D mobile platform, and charge-coupled device camera.

The projection optics consist of a plano-concave lens, plano-convex lens, and 10× ultraviolet (UV) objective lens [1]. Among them, the digital micromirror device is the core component of the system and provides light modulation. The UV light is irradiated to the surface of the digital micromirror device through the beam expander. The digital micromirror device is utilized as a dynamic mask to modulate the UV light. The device can accurately reflect the designed pattern to the projection optics by adjusting the deflection of micromirrors, as shown in Figure S1.

The DMD is composed of 1024 × 768 miniature reflective mirrors, each with a size of 13.6 × 13.6 μm. The exposure area of DMD is

$$S = M \times 13.6 \times 13.6 \times |\beta| \quad (1)$$

where M is the number of micromirrors, and β is the zooming times of the objective lens group.

In this case, the approximate resolution of the entire system is obtained as follows:

$$R = 13.6 \times |\beta| \quad (2)$$

Because a 10× objective lens is used, an approximate resolution of 1.36 μm can be obtained [1].

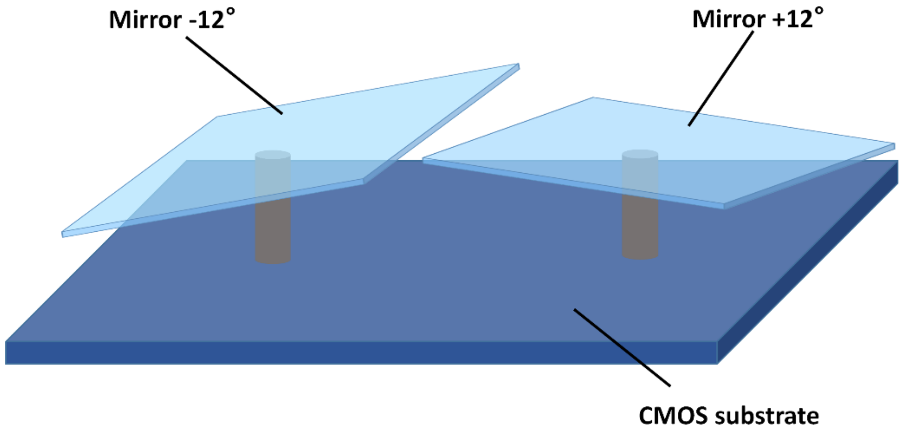

Figure S1. Schematic of digital micromirrors.

In the experiments, a UV laser with a wavelength of 365 nm and a maximum power of 53.33 mW/cm^2 was used. The Leica vz105 series objective lens used had a magnification factor of 140 and numerical-aperture value of 0.28. Generally, the maximum exposure size was 800 × 800 μm. During each single exposure, 70 microwells with diameters of 30 μm could be produced.

The experimental and simulation results reveal that it is easier to capture the small sphere at a depth of approximately 30 μm. The depth of a microwell array in the capturing cell experiments was

approximately 30 µm. As shown in Figure S2, when the exposure duration time was up to 9 s, the depth of the microwells remained unchanged. Therefore, in most experiments, the exposure duration time was set to 5 s. The average depth of the microwell array was approximately 30 µm.

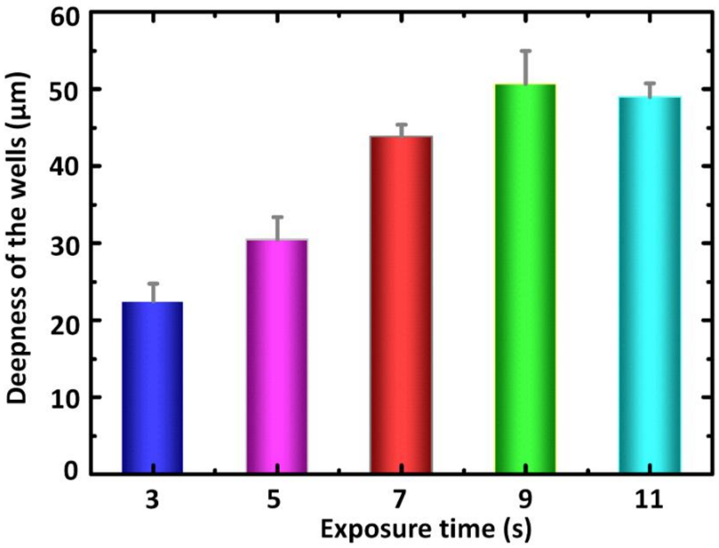

Figure S2. Relation between deepness of the microwells and exposure time.

When the microwell arrays were fabricated, the depth of the microwell array was measured by a HIROX microscope (Hirox Asia Ltd., Hong Kong, China). The SEM image in Figure S3 shows that the shape of the manufactured microwell array was consistent with the designed structures.

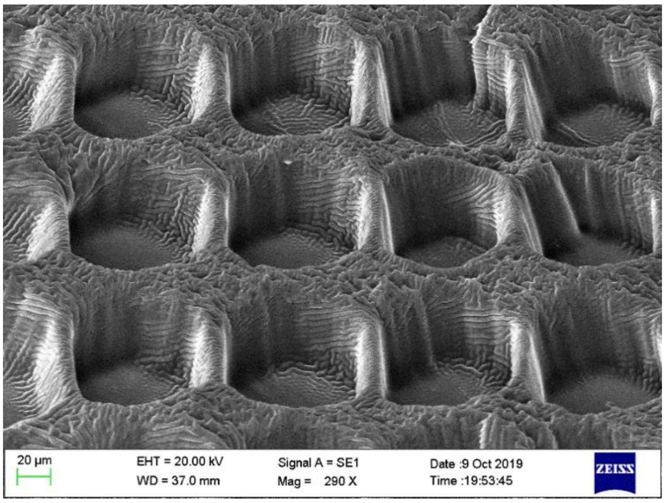

Figure S3. SEM image of microwell array.

2. Theoretical Analysis

In a fluid condition, the fluid resistance must be considered. Stokes forces cannot be ignored, and the Magnus forces can be ignored.

The situation of the microspheres captured in the experiments shows that the position where the microspheres and cells were captured is usually in the middle of the microwells. Meanwhile, according to the simulation results, regardless of the pattern of the microwell array, the flow field velocity is larger in the central portion. There are two general cases where particles pass through the microwell array. As shown in Figure 7, the blue particles flowed to the edges of the microwell, and

yellow particles flowed to the middle portion of the microwells. When the particles flowed to the edge of the microwells, as in Figure 7c, the gravity was equal to the buoyancy. When the particles flowed through the edge of the microwells, the Stokes force was not negligible. The particles were spherical, and the fluid had a flow velocity. The Stokes force is

$$F_{\mathrm{D}} = 6\pi\mu R\Delta V \quad (3)$$

$$\Delta V = V - V_{\mathrm{P}} \quad (4)$$

R represents the particle radius, and μ is the liquid viscosity coefficient. V and V_P represent the velocities of the fluid and the particles, respectively. The direction of the Stokes force is the same as the velocity vector difference. The above analysis demonstrates that the particle easily flows out of the microwells under the action of the final acceleration. The particles are accelerated to move in the liquid. The accelerated surrounding fluid can generate an additional mass force Fi. Finally, the Stokes force, gravity force, and buoyancy force work together to make the particles move toward the bottom of the microwells. In addition, the particle velocity at the bottom of the microwells is gradually reduced, and the particles settle to the bottom of the microwells by the gravity force.

In simulation, the N–S equation is simplified as follows:

$$\frac{\partial V}{\partial t} + (V \cdot \nabla)V = -\frac{1}{\rho}\nabla p + (\mu\nabla^2 V + f_\vartheta) \quad (5)$$

In general, the first term on the left side of the equation indicates local acceleration, and the second term on the left side is the convection term. The first term on the right side is the pressure gradient, and the second term on the right side is the diffusion term.

The steady laminar flow between the microwells performed can be calculated from the N–S equation. The boundary conditions, pressure, material, and geometry can be set. According to the equation, the pressure at any point can be predicted. The velocity distribution, average velocity, and flow rate can be simulated.

The original formula is as follows:

$$f_\vartheta = \frac{\partial^2 V}{\partial y^2} \quad (6)$$

Reference

1. Yang, W.G.; Yu, H.B.; Li, G.X.; Wang, Y.C.; Liu, L.Q. High-throughput fabrication and modular assembly of 3D heterogeneous microscale tissues. *Small* **2017**, *13*, 1–11.

© 2019 by the authors. Submitted for possible open access publication under the terms and conditions of the Creative Commons Attribution (CC BY) license (http://creativecommons.org/licenses/by/4.0/).
